# Studies on the Japanese soil-borne wheat mosaic virus movement protein highlight its ability to bind plant RNA

**DOI:** 10.1186/s12985-025-02757-z

**Published:** 2025-05-07

**Authors:** Claudia Janina Strauch, Nico Sprotte, Estefania Peña Lozano, Emmanuel Boutant, Khalid Amari, Steffen Ostendorp, Anna Ostendorp, Julia Kehr, Annette Niehl

**Affiliations:** 1 Institute for Epidemiology and Pathogen Diagnostics, Julius Kühn Institute (JKI) - Federal Research Centre for Cultivated Plants, Messeweg 11-12, 38104 Brunswick, Germany; 2 Institute of Plant Science and Microbiology, University of Hamburg, Ohnhorststr. 18, Hamburg, 22609 Germany; 3 Laboratory of Bioimaging and Pathologies, CNRS UMR 7021, Faculty of Pharmacy, University of Strasbourg, 74 Route du Rhin - CS 60024, F-67400 Illkirch Strasbourg, France; 4 Institute for Biosafety in Plant Biotechnology, Julius Kühn Institute (JKI) - Federal Research Centre for Cultivated Plants, Erwin-Baur-Str. 27, 06484 Quedlinburg, Germany; 5 Present Address: Biotechnology and Cell Signaling, CNRS UMR7242, ESBS, University of Strasbourg, Bld Sébastien Brant, F-67412 Illkirch Strasbourg, France

**Keywords:** Furovirus, Virus-host interaction, RNA binding, Movement protein function

## Abstract

**Background:**

Plant viral movement protein (MP) function is decisive for virus cell-to-cell movement. Often, MPs also induce membrane alterations, which are believed to play a role for the establishment of viral replication compartments. Despite these central roles in virus infection, knowledge of the underlying molecular mechanisms by which MPs cause changes in plasmodesmata (PD) size exclusion limit and contribute to the formation of viral replication compartments remain far from being complete.

**Methods:**

To further identify host processes subverted by viral MPs, we here characterized the MP of Japanese soil-borne wheat mosaic virus (JSBWMV). We used confocal fluorescence microscopy to study the subcellular localization of MP^JSBWMV^ and to address its functionality in promoting virus cell-to-cell movement. Using the biochemical and biophysical methods co-immunoprecipitation, fluorescence lifetime imaging, microscale thermophoresis and RNA immunoprecipitation we investigate the capacity of MP^JSBWMV^ to multimerize and to bind viral and cellular RNAs.

**Results:**

MP^JSBWMV^ localized to PD, promoted cell-to-cell movement by complementing a movement-deficient unrelated virus, formed multimers *in-vivo* and bound to viral RNA with high affinity. Using RNA immunoprecipitation, we identified host RNAs associated with the viral MP. Within the MP-RNA complexes we found RNAs encoding proteins with key functions in membrane modification, signaling, protein folding, and degradation. We propose that binding of MP to these RNAs during infection and regulation of their spatio-temporal translation may represent a mechanism for MPs to achieve PD and host control during replication and movement.

**Conclusion:**

This study provides new insight into the complex interactions between viral MPs and host cellular processes.

**Supplementary Information:**

The online version contains supplementary material available at 10.1186/s12985-025-02757-z.

## Introduction

Plant viruses usually encode movement proteins (MPs) which are essential for virus movement between cells. These MPs facilitate movement of the plant viral genome as viral ribonucleoprotein complex or as virus particle through plasmodesmata (PD), the symplasmic connection channels between plant cells. In order to function as viral MP, they interact with the viral genome, target it to PD, and either regulate the PD size exclusion limit (SEL) or modify PD to form tubules inside the channels. Thus, characteristic features of viral MPs are their localization to PD, interaction with host and viral proteins including self-interaction, and the ability to bind nucleic acids [1–3].

Different families of MPs have been identified in plant viruses. The most prominent MP family is the 30 K MP-family, including the tobacco mosaic virus (TMV) MP [4], that modify the PD SEL. Such viral MPs regulate PD SEL indirectly by interacting with host factors that directly or indirectly affect cellular processes leading to PD SEL alteration, such as callose deposition or degradation at the PD pore. Moreover, apart from realizing transport of the viral genome between cells, MPs also move non-cell autonomously between cells. These capacities have led to hypotheses that MPs may act as “conditioners” to create a favorable environment ahead of infection by modulating plant processes affecting PD SEL and the translation of proteins [5]. Also endogenous RNA and protein signals during development and defense require control of PD permeability and transport between cells [6, 7]. Thus, a detailed understanding of MP function with respect to the modulation of host processes by binding endogenous RNAs and proteins is of central importance for understanding the regulation of macromolecular transport in plants.

Furoviruses belong to the family of *Virgaviridae* and are transmitted by the obligate root-parasite *Polymyxa graminis* [8–10]. The furovirus Japanese soil-borne wheat mosaic virus (JSBWMV) consists of a positive-stranded bipartite RNA genome encapsidated separately in rigid rod-shaped particles [11]. The JSBWMV RNA genome possesses a 5’- terminal cap (m7GpppG) structure and a 3’-terminal tRNA-like structure [12, 13]. The RNA1 is about 7.2 kb long and contains two open reading frames. The first open reading frame encodes a replicase with a methyltransferase and helicase domain, while a larger replicase protein is produced via translational read-through of the stop codon of the first replicase gene. This results in a replicase protein which contains an additional RNA-dependent RNA-polymerase domain [11]. RNA1 also encodes the putative 37 kDa movement protein (MP) [11, 14]. RNA2 with a length of 3.6 kb encodes four proteins, among them the capsid protein (CP), a CP-readthrough protein presumably involved in virus transmission, and a silencing suppressor [11, 15–18]. The MPs of the furoviruses soil-borne wheat mosaic virus (SBWMV) and Chinese wheat mosaic virus (CWMV) have been studied before [14, 19] Both MPs were shown to localize to PD, thus to exhibit a typical localization for MPs. Targeting of MP^CWMV^ to PD was shown to depend on the secretory pathway.

To gain additional insight into JSBWMV infection and the role of its MP in particular, we here investigated properties of the MP^JSBWMV^. We examined the subcellular localization of a GFP-tagged MP during virus infection, as well as when expressed ectopically in *N. benthamiana* host cells. Additionally, we assessed whether the tagged MP could facilitate movement of a movement-deficient TMV. As a common feature of MPs, we confirmed interaction of MP^JSBWMV^ with itself and explored binding of MP^JSBWMV^ to the viral RNAs. To gain insight into host processes modulated by the viral MP^JSBWMV^, we focused on cellular RNAs that can interact with the MP^JSBWMV^, as identified through RNA immunoprecipitation assays.

## Methods

### Plant material

*Nicotiana benthamiana* and *N. tabacum* cv. Xanthi line 277 expressing MP^TMV^ [20] were grown at 20–24 °C with 16 h light and 8 h dark cycles and at least 200 W/m^2^ light intensity in the greenhouse. As efficient replication of JSBWMV in cells requires 17 °C (Fig S1 [21]), plants infected with JSBWMV were kept at 17 °C with 16 h light and 8 h dark cycles and at least 200 W/m^2^ light intensity.

### Generation of constructs

MP^JSBWMV^:RFP and MP^JSBWMV^:GFP binary vectors were generated by GATEWAY cloning according to the manufacturer’s instructions [22] (Invitrogen, Thermo Fisher Scientific, Waltham, USA). The open reading frame of MP was PCR-amplified from the pJS1 plasmid, carrying the cDNA-sequence from JSBWMV RNA1 [23] and recombined into pDONR™/Zeo (Invitrogen). For the N-terminal fusion of MP^JSBWMV^ to GFP or RFP the destination vectors pB7FWG2 or pH7RWG2 were used, respectively [22]. To generate the binary vector expressing free GFP, the eGFP sequence was PCR-amplified from pB7 FWG2 using primers containing Gateway recombination sites and recombined into pDONR™/Zeo. Subsequently, the entry clone was recombined into the pGWB2 destination vector. DNA sequencing verified the inserts of entry and expression vectors. MP^JSBWMV^:GFP was cloned into a GoldenGate compatible pET28a + bacterial expression vector. MP^JSBWMV^:GFP was fused with an N-terminal 6xHisTag followed by a thrombin cleavage site. MP^JSBWMV^:GFP was amplified with primers harboring *Bsa I* cleavage sites. GoldenGate reaction was carried out in 50 cycles by incubation at 37 °C for 2 min followed by incubation at 16 °C for 5 min with T4 ligase (ThermoFisher Scientific) and *Bsa I* (New England Biolabs (NEB), Ipswich, USA). After a subsequent incubation at 37 °C for one hour, T4 ligase and *Bsa I* were inactivated at 80 °C for 10 min. All primers are depicted in Table S1. All other binary vectors were published before: PDLP1:RFP [24], PDLP1:GFP [25], VAP27:GFP [26], AtREM1.2:CFP, AtREM1.3:CFP, AtREM6.1:CFP, AtREM6.2:CFP, AtREM6.3:CFP, AtREM6.4:CFP [27], pGWB455 (free RFP, cytoplasmic marker [28]) and P19 [29].

### Transient expression of proteins by agro-infiltration

For expression of MP^JSBWMV^:GFP/RFP the binary constructs were transformed into the agrobacterium strain GV3101. Agrobacteria C58C1 containing P19 were co-infiltrated in all samples. Agrobacteria were grown in 5 mL 2 × yeast-tryptone-media at 28 °C containing selective antibiotics. For inoculation, the bacteria were harvested and resuspended in buffer (10 mM MES (2-(N-morpholino)ethanesulfonic acid), 10 MgCl_2_). The bacterial density at OD_600_ was adjusted to 0.3 for each construct-carrying strain when two constructs were co-inoculated, 0.5 for inoculation of one construct-carrying agrobacteria strain, and 0.1 for P19. The agrobacteria mixtures were infiltrated into the abaxial side of *N. benthamiana* leaves using a syringe without needle.

### Modification of JSBWMV cDNA-clone

The cDNA-clones pJS1 and pJS2 [23] were published before. To generate pJS1-MP:RFP, where the MP is expressed as fusion to the N-terminus of RFP, a partial MP:RFP sequence was PCR amplified from MP^JSBWMV^:RFP and *Bgl*II restrictions sites were added (Primer see Tab S1). The PCR products from MP^JSBWMV^:RFP and pJS1 were digested using *Bgl*II (NEB). *Bgl*II cuts pJS1 at nucleotide position 6447. Digested DNAs were purified with NucleoSpin Gel and PCR Clean-up kit (Macherey–Nagel, Düren, Germany). The partially digested pJS1 and MP^JSBWMV^:RFP were then ligated using T4 DNA Ligase (NEB). Modified pJS1-MP:RFP was propagated in *E. coli* MC1061 and sequence validation was performed by sequencing.

### RNA synthesis

Prior to RNA synthesis, plasmids were multiplied in *E. coli* MC1061 in LB-media with the corresponding antibiotics (for TMV∆MP∆CP-GFP 20 mM sucrose was added to the media). RNA was synthesized from plasmid templates or PCR-purified templates. The plasmids pJS1, pJS1-MP:RFP and pJS2 were linearized with *Spe*I-HF® (NEB) and TMV∆MP∆CP-GFP was linearized with *KpnI* (Thermo Fisher Scientific) and subsequently purified with the NucleoSpin Gel and PCR Clean-up kit (Macherey–Nagel) according to the manufacturer’s protocol before use as RNA-synthesis template. RNA synthesis of pJS1, pJS1-MP:RFP and pJS2 was performed using the SP6 promoter present in the plasmids and the SP6 RiboMAX™ Large Scale RNA Production System (Promega, Madison, USA) according to the manufacturer’s instructions with minor changes. TMV∆MP∆CP-GFP RNA was synthesized using the T7 RiboMAX™ Large Scale RNA Production System. For MST analyses, RNA2^JSBWMV^, a part of RNA1^JSBWMV^ or RNA2^JSBWMV^ containing the tRNA-like structure, and a part of RNA1^JSBWMV^ or RNA2^JSBWMV^ without the tRNA-like structure were PCR-amplified by including a T7-promotor for RNA-synthesis. PCR was conducted using pJS1 and pJS2 as templates (primers are depicted in Table S1). PCR-amplified RNA2, tRNA-like structures, and non-tRNA-like structures harboring a T7-promotor were used to synthesize RNA with the T7 RiboMAX™ Large Scale RNA Production System (Promega). In all RNA synthesis systems the rNTP mix was changed to a concentration of 5 mM ATP, CTP, UTP and 0.6 mM GTP in the reaction mix. The m7G cap analogue (2.67 mM m_2_^7.3´−O^GP3G (ARCA) Cap-analog solution; Jena Bioscience, Jena, Germany) was used in RNA synthesis. In RNA synthesis reactions for MST analyses, 5% DMSO was added to the RNA synthesis reaction mix. RNA used in MST was cleaned from the DNA template by applying RNase-free DNase (Promega) according to the manufacturer’s protocol followed by a column-based RNA isolation (RNA Clean & Concentrator-25, Zymo Research, Irvine, USA).

### Virus inoculation

Transcribed RNA1^JSBWMV^^-MP:RFP^ and RNA2^JSBWMV^ were mixed equally and diluted 1:4 with inoculation buffer (50 mM glycine; 50 mM K_2_HPO_4_ (pH 9.2)). Transcribed RNA^TMV∆MP∆CP−GFP^ was diluted 1:9 with sterile distilled water. Leaves of *N. benthamiana* (2–4 weeks old) were powdered with celite. RNA was added as a drop on the upper side of the leaves. The RNA was then inoculated by carefully rubbing of the leaf surface. After 20 min the leaves were rinsed with water.

### Movement complementation assay

Ability of MP^JSBWMV^:RFP to facilitate movement of a movement-deficient TMV was tested. TMV∆MP∆CP-GFP [30] was published before. MP^JSBWMV^:RFP or free RFP were expressed for three days in *N. benthamiana* before RNA^TMV∆MP∆CP−GFP^ was inoculated. Sizes of the infection sites were analyzed after four days. As control that TMV∆MP∆CP-GFP movement could be complemented by the provision of an MP in trans, *N. tabacum* cv. Xanthi line 277 expressing MP^TMV^ [20] was inoculated with RNA^TMV∆MP∆CP−GFP^ and infection sites analyzed by fluorescence microscopy after three days.

### Fluorescence Microscopy and image processing

Localization of MP^JSBWMV^ was analyzed after 2 to 5 days after agro-inoculation. Aniline blue staining was performed with aniline blue solution (67 mM sodium phosphate, 0.5–1% aniline blue) infiltrated in leaf discs using a vacuum pump. The samples were kept in a dark place for ten minutes before being analyzed by confocal microscopy. A CLSM platform with a Leica DM6 microscope (Leica microsystems, Wetzlar, Germany) and the TCS SP8 multiphoton system was available. Microscopic observations were performed with the objectives HC PL FLUOTAR 10x/0.32 dry, HC PL APO 20x/0.75 IMM CORR CS2 and HCPL APO 63x/1.20 CORR CS2 water immersions in combination with an Acousto-Optical Beam Splitter for detection. Aniline blue and CFP fluorescence was detected between 420–480 nm with excitation using a 405 nm diode. GFP fluorescence was detected between 495 and 555 nm by excitation with a 488 nm argon laser. RFP fluorescence was detected between 590–630 nm and was excited with a 561 nm diode pumped solid state laser. The images and processing were performed with Leica Application Suite X software (version: 3.5.7.23225, Leica microsystems). The pinhole was kept at airy unit = 1.0.

### Protein detection in western blots

Plant samples or precipitated proteins from IP were mixed with SDS-sample buffer (120 mM TRIS–HCl (pH 6.8), 20% glycerol, 4% SDS, 0.04% bromophenol blue, 10% β-mercaptoethanol) and prior to loading to a SDS-PAGE. SDS-PAGE was performed with 12% separating gel and 6% stacking gel followed by electro blotting onto an Immobilon P PVDF membrane (0.45 μm pore size, Millipore, Merck, Sigma-Aldrich, St. Louis, USA). GFP was detected with the primary antibody GFP polyclonal antibody (PA1-980 A, Thermo Fisher Scientific) and the Goat anti-Rabbit IgG (H + L), horseradish peroxidase conjugated secondary antibody (Thermo Fisher Scientific). RFP was detected with the primary antibody RFP monoclonal antibody (RF5R, Thermo Fisher Scientific) and the F(ab')2-Goat anti-Mouse IgG (H + L) secondary antibody, horseradish peroxidase (Thermo Fisher Scientific). The PageRuler™ Plus Prestained Protein Ladder, 10 to 250 kDa (Thermo Fisher Scientific) was used to determine protein sizes. Peroxidase signal was detected using SuperSignal™ West Pico PLUS Chemiluminescent Substrate (Thermo Fisher Scientific).

### Co-immunoprecipitation

*N. benthamiana* leaves expressing fluorescent proteins were used for IPs to analyze protein–protein interactions. For the IPs, RFP-Trap® magnetic agarose beads (Chromotek, Proteintech, Chicago, USA) were used according to the manufacturer’s protocol with minor changes. The lysis buffer contained 50 mM TRIS–HCl (pH 7.5); 150 mM NaCl; 2.5 mM MgCl_2_; 0.5% Nonidet® P40 (Substitute) BioChemica (AppliChem GmbH, Darmstadt, Germany); 1 × Protease Inhibitor cocktail (cOmplete Tablets Mini, EDTA-free; Roche, Basel, Switzerland). The proteins were eluted by adding 2 × SDS-sample buffer according to the manufacturer’s instructions.

### Fluorescence lifetime imaging microscopy (FLIM)

Fluorescence resonance energy transfer (FRET)-FLIM experiments were performed as described in [31] and [32]. Shortly summarized, time-correlated single-photon counting FLIM measurements were accomplished with a home-built two-photon system. An Olympus IX70 inverted microscope with an Olympus 60 × 1.2 NA water immersion objective was utilized as basis. For two-photon excitation, a mode-locked titanium:sapphire laser (Tsunami, Spectra Physics, http://www.newport.com) was utilized with an emission wavelength of 900 nm. The pile-up effect was avoided by adjusting the laser power to give counting rates with peaks up to a few 100 photons sec^−1^. The laser scanning system operating with two fast galvo mirrors (Model 6210; Cambridge Technology, http://www.camtech.com) was used to perform imaging. For this purpose, the laser scanning system worked in the descanned fluorescence collection mode. The collection of photons was performed with a two-photon short-pass filter with a cut-off wavelength of 680 nm (F75–680; AHF, http://www.ahf.de), and a band-pass filter of 520 ± 17 nm (F37–520; AHF). Connected to a time-correlated single photon-counting (TCSPC) module (SPC830; Becker & Hickl, http://www.becker-hickl.de), operating in a reversed start-stop mode, a fiber-coupled avalanche photodiode (SPCM-AQR-14-FC; Perkin Elmer, http://www.perkinelmer.com) was used for fluorescence detection.

To gain an appropriate photon statistic for the fluorescence decays, the samples were scanned continuously for 30 s to 120 s. The time was adjusted to collect a sufficient number of photons for each sample. Data analysis was performed with the software package (SPCIMAGE V2.8; Becker & Hickl), which applied an iterative reconvolution method to recover the lifetimes from the fluorescence decays. The following formula was used to calculate the FRET-efficiency E.

$$E=\left(\frac{{R}_{0}^{6}}{{R}_{0}^{6}+R}\right)=1-\frac{{\tau }_{fret}}{{\tau }_{free}}$$

In this formula, R_0_ represents the Förster radius, R is the distance between the donor and the acceptor, τ_fret_ reflects the lifetime of the donor in the presence of the acceptor, τ_free_ is the lifetime of the donor in the absence of the acceptor.

To calculate the FRET efficiency in the different samples, the lifetimes of the donor fluorophore were measured. For this purpose, under “options” “model” the setting “incomplete multiexponentials” was chosen. The borders of the decay curve were manually adjusted. Fluorescent spots with more then 10 000 pixel and Χ2-value (indicator of the fit quality of the decay curve) between 1 and 2 were used for lifetime measurement. With a FRET-efficiency value above 5%, it was considered that protein–protein interaction occurs between the donor and the acceptor.

### RNA binding quantification using microscale thermophoresis (MST)

RNA binding quantification of plant leaf extracts was made with extracts from agroinfiltrated *N. benthamiana* leaves transiently expressing MP^JSBWMV^:GFP or free GFP for 3 to 4 days. Leaf extracts were prepared as described previously with minor modifications [33, 34]. In addition to leaf extract, MP^JSBWMV^:GFP purified from *E. coli* BL21 + RIPL was used in MST. Bacteria carrying the MP^JSBWMV^:GFP plasmid were grown in 50 mL autoinduction medium containing kanamycin (200 µg/mL) at 24 °C overnight followed by protein extraction. Cells were lysed in 5 mL of lysis buffer (50 mM Tris–HCl pH 8.0, 150 mM NaCl, 5 mM DTT supplemented with one protease inhibitor tablet (complete protease inhibitor cocktail, (Merck) and 1 × BugBuster) followed by a 30 min incubation on ice. MP^JSBWMV^:GFP was purified using a GST-tagged antiGFP-nanobody (Addgene No. #61,838) coupled to magnetic glutathione beads (Millipore, Merck). In brief, 200 µg purified GST-antiGFP-nanobody was incubated with 30 µL of magnetic beads, equilibrated in lysis buffer. After subsequent washing, coupled beads were added to the *E.coli* cell lysate and incubated for one hour on ice. Bound MP^JSBWMV^:GFP was washed three times with 1 mL lysis buffer and subsequently eluted with lysis buffer supplemented with 10 mM glutathione. Glutathione was removed via dialysis against lysis buffer.

For MST in leaf extracts, leaf material was ground in liquid nitrogen using mortar and pestle. 200 µL of 2 × MST buffer (100 mM Tris–HCl pH 7.5, 300 mM NaCl, 10 mM MgCl_2_, 0.1% (v/v) Tween-20, 0.1 mg/mL BSA, 1 × protease inhibitor mix (complete protease cocktail, Merck), 10 mM DTT) was added per 100 mg of ground leaf material, incubated on ice for 30 min and subsequently centrifuged at 20 000 × *g* for 10 min at 4 °C. Centrifuged supernatants were collected and further diluted until fluorescence counts between 400 and 1000 were achieved. For RNA binding quantification, serial dilutions of target RNAs were made and assays were performed according to the manufacturer's instructions. Samples were measured in standard capillaries on a Monolith NT.115 (NanoTemper GmbH, Munich, Germany) with medium MST power and analyzed using MO. Affinity Analysis software. Binding was regarded as true when a signal-to-noise ratio and response amplitude larger than 5 was achieved as suggested by the manufacturer [33, 34]. Binding curves obtained in MST analysis are presented in Fig S2.

### RNA immunoprecipitation (RIP)

RIP was conducted with leaves transiently expressing MP^JSBWMV^:GFP or GFP as control. To increase the amount of RNA in RIP samples, a formaldehyde fixation was applied, in which 1% formaldehyde-solution was infiltrated into leaves, followed by a washing step with infiltration of glycine-solution (125 mM) [35]. Leaves were rinsed with ice-cold dH_2_O before being frozen in liquid nitrogen. Three leaves from independent plants were pooled for RIP. 500 mg leaf material was used for RIP with GFP-Trap® magnetic agarose beads (Chromotek) according to the manufacturer’s protocol with minor changes. The lysis buffer contained 50 mM TRIS–HCl (pH 7.5); 150 mM NaCl; 2.5 mM MgCl_2_; 0.5% Nonidet® P40 (Substitute) BioChemica (AppliChem GmbH); 1 × Protease Inhibitor cocktail (cOmplete Tablets Mini, EDTA-free; Roche); 50 U/mL RNase inhibitor (murine, NEB); 0.5 mM DTT). TRI Reagent® (Sigma-Aldrich) was added to the beads for elution of proteins. RNA extraction was performed according to the manufacturer’s instructions. Standard RNA-Seq was performed on an Illumina NovaSeq platform (Genewiz, Azenta, Chelmsford, USA) with a depth of 10 million read pairs per sample in a 2 × 150 bp configuration. After Sequencing, two replicates from MP^JSBWMV^:GFP and GFP RIP, respectively, showed high quality for bioinformatic analyses. RNA-Seq data were analyzed with the Galaxy platform [36, 37]. Removing of adapters and trimming (reads shorter than 20 nt) was performed with TrimGalore. Reads were mapped to *N. benthamiana* reference dataset (https://solgenomics.net/) using the “hierarchical indexing for spliced alignment of transcripts” (HISAT) program [38] and counted with Samtools idxstats [39]. Venn diagram comparison was used to identify common and specific genes among the different treatment [40]. Counts per million for each replicate was used to calculate the log2 fold change between MP^JSBWMV^:GFP replicates and GFP replicates. RNAs with a fold change over four was used for further interpretation (Table 2), where the solgenomics annotation and the proteins-sequence were used to identify known function for these RNA/proteins in databases.

## Results

### MP^JSBWMV^ localizes to PD and plasma membrane microdomains

**Fig. 1:**
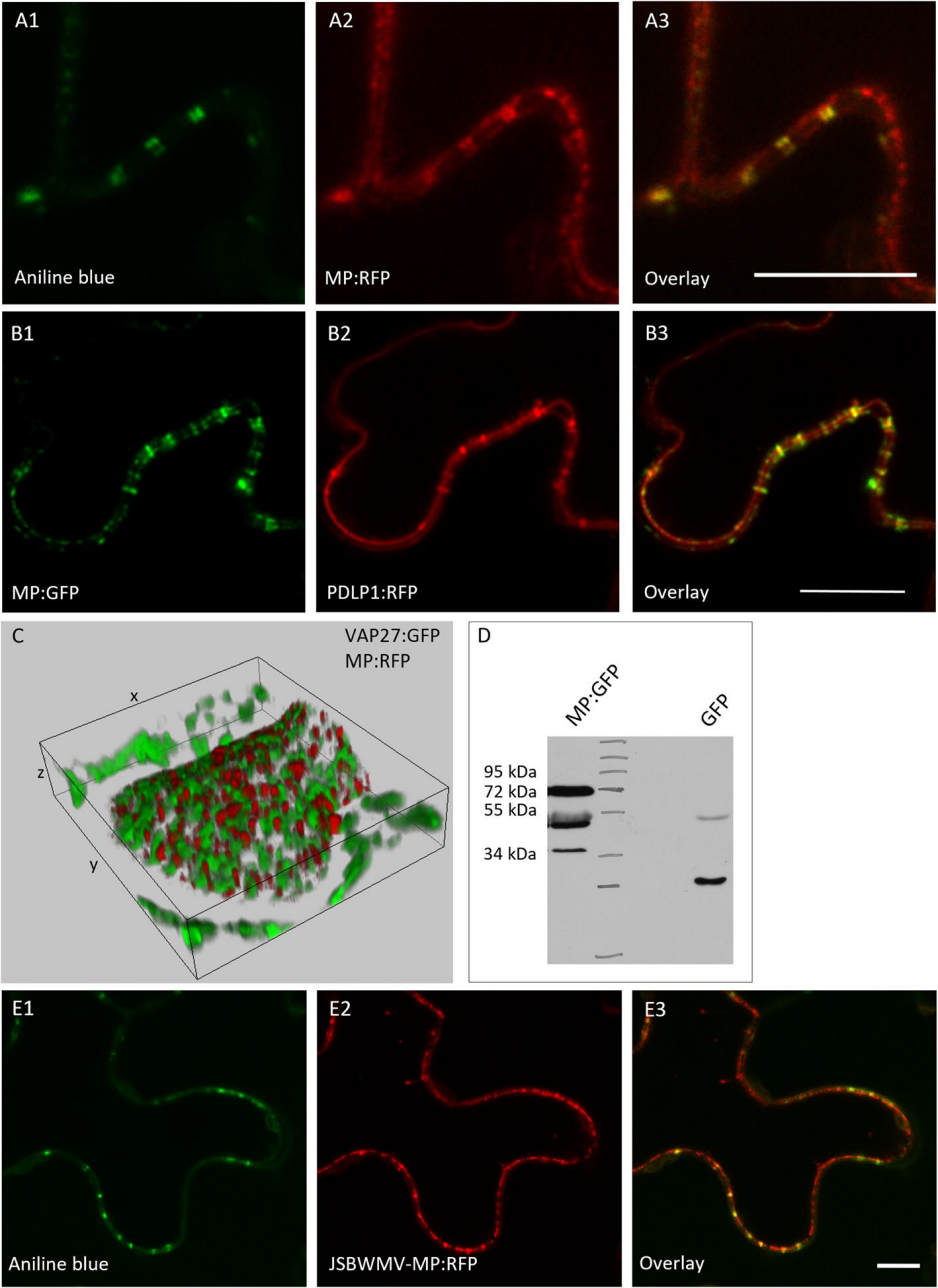
Subcellular localization of MP^JSBWMV^ in *N. benthamiana* epidermis cells. **A-B** MP^JSBWMV^ was ectopically expressed in *N. benthamiana* epidermal cells via agroinoculation and co-expressed with different markers. (**A**) cells stained with aniline blue (A1), expression of MP^JSBWMV^:RFP (A2) and co-localization of the aniline blue signal and MP^JSBWMV^:RFP signal in the overlay (A3); (**B**) MP^JSBWMV^:GFP (B1) co-expressed with PDLP1:RFP (B2); MP^JSBWMV^:GFP co-localizes with the PD-marker PDLP1:RFP in PD (B3); (**C**) MP^JSBWMV^:RFP (red) co-expressed with the marker for ER-plasma membrane attachment sites VAP27-GFP (green) z-stack (13 slices, z: 4.27 μm, y: 34.16 μm, x: 30.65 μm). The proteins localize in a patchwork pattern in the cell cortex. Images taken two to five days post inoculation of agrobacteria. Scale bars are 10 μm; (**D**) Western blot showing MP^JSBWMV^:GFP at approximately 70 kDa and GFP at approximately 27 kDa using a specific antibody against GFP and peroxidase-labeled secondary antibodies. Bands at appoximatey 50 and 38 kDa likely represent degradation products of MP^JSBWMV^:GFP. The bands at approximately 55 kDa likely represent a cross reaction of the antibody with the large subunit of RuBisCo. **E** cells stained with aniline blue (E1) in JSBWMV-MP:RFP infection sites 13 days after inoculation of RNA^JSBWMV−MP:RFP^ (E2) and overlay of aniline blue and MP^JSBWMV^:RFP showing the localization to PD (E3)

To investigate the subcellular localization of MP^JSBWMV^ in plant cells, we constructed the fusion proteins MP^JSBWMV^:GFP and MP^JSBWMV^:RFP for ectopic transient expression in *N. benthamiana* epidermal cells using agro-infiltration. In co-localization studies with the PD marker PDLP1:RFP and callose staining at PD using aniline blue, we confirmed the localization of MP^JSBWMV^:GFP and MP^JSBWMV^:RFP at PD (Fig. 1 A-B). Additionally, MP^JSBWMV^:GFP and MP^JSBWMV^:RFP localized at plasma membrane (PM) microdomains as indicated by localization with an ER-plasma membrane-contact-sites marker VAP27:GFP [26] (Fig. 1C). Localization of MP^JSBWMV^:RFP in rafts at the plasma membrane was also confirmed by localization studies with the *Arabidopsis thaliana* remorins REM1.2:CFP, REM1.3:CFP, REM6.1:CFP, REM6.2:CFP, REM6.3:CFP and the REM6.4:CFP [27] (Fig S3 A-F). Although none of the used PM markers co-localized with the MP^JSBWMV^:RFP, fluorescence appeared in a patchy pattern in the same cortical layer. The ectopically expressed fusion protein MP^JSBWMV^:GFP, with a size of approximately 70 kDa, was detected and confirmed by western blot using crude plant extracts (Fig. 1 D). To investigate MP^JSBWMV^ localization during infection, we modified the pJS1-clone harboring the cDNA-sequence of a Japanese isolate of JSBWMV [23] to express a fusion of the MP to RFP. Infectious RNA was *in-vitro* synthesized and RNA^JSBWMV−MP:RFP^ was rub-inoculated onto *N. benthamiana* leaves. The JSBWMV-MP:RFP was able to initiate multicellular infection sites on *N. benthamiana* leaves, showing that the RFP-modification did not abolish virus infectivity and cell-to-cell movement (Fig S3 G). Furthermore, we verified the localization of the MP^JSBWMV^:RFP to PD in viral infection sites by co-localization with aniline blue (Fig. 1 E).

### MP^JSBWMV^ can complement the movement of a movement-deficient TMV

**Fig. 2:**
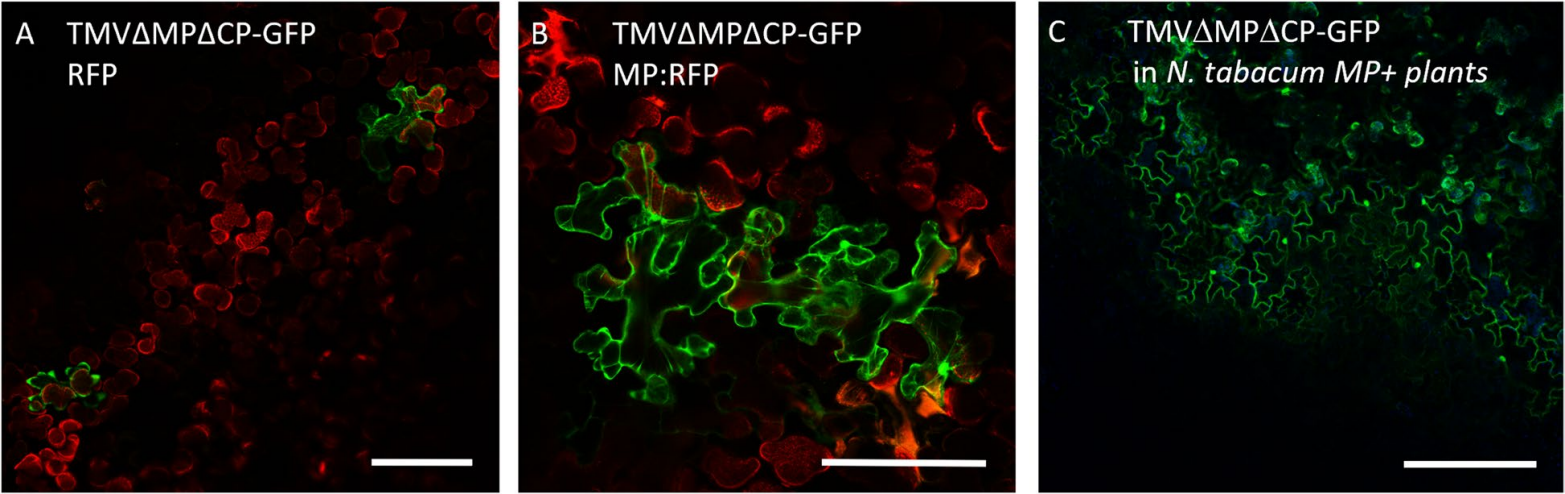
MP^JSBWMV^:RFP complements TMVΔMPΔCP-GFP for movement. Infectious RNA was synthesized from TMVΔMPΔCP-GFP and rub inoculated into *N. benthamiana* leaves expressing free RFP (**A**) or MP^JSBWMV^:RFP (**B**) for three days. (**C**) To control that TMVΔMPΔCP-GFP movement can be complemented by a functional MP, TMVΔMPΔCP-GFP was inoculated onto *N. tabacum* cv. Xanthi line 277 plants constitutively expressing the TMV MP (MP +, [20]). Pictures were taken four days (**A**, **B**) or three days (**C**) after inoculation of TMVΔMPΔCP-GFP. Scale bars are 200 µm

Localization to PD and the formation of multicellular JSBWMV-MP:RFP infection sites suggested that the fluorescent protein-fused MP^JSBWMV^ was functional in facilitating cell-to-cell movement. To confirm this, we performed a complementation assay with a movement-deficient, infectious TMV. This TMV expressed GFP from the CP promoter and contained a truncated MP sequence (TMVΔMPΔCP-GFP) [30]. To test if the ectopically expressed MP^JSBWMV^ was able to restore movement of TMV∆MP∆CP-GFP, we expressed either MP^JSBWMV^:RFP or RFP (as a control) in *N. benthamiana* leaf cells. Three days post inoculation, infectious RNA^TMVΔMPΔCP−GFP^ was rub-inoculated into these leaves. After four days, leaves were screened at the fluorescence microscope for multicellular infection sites. In leaves expressing RFP, only single TMVΔMPΔCP-GFP infected cells were observed (Fig. 2A), while in leaves expressing MP^JSBWMV^:RFP multicellular TMVΔMPΔCP-GFP infection sites were visible (Fig. 2B). To further confirm that MP provided in trans is responsible for the formation of multicellular infection sites, we also infected *N. tabacum* cv. Xanti line 277 plants constitutively expressing the TMV MP [20] with TMVΔMPΔCP-GFP and imaged the infection sites formed from three days after infection by fluorescence microscopy (Fig. 2C). This demonstrates the ability of MP^JSBWMV^ to complement the movement of TMVΔMPΔCP-GFP, further confirming that the fluorescent tag did not abolish MP function to facilitate cell-to-cell spread.

### MP^JSBWMV^ can self-interact

**Fig. 3:**
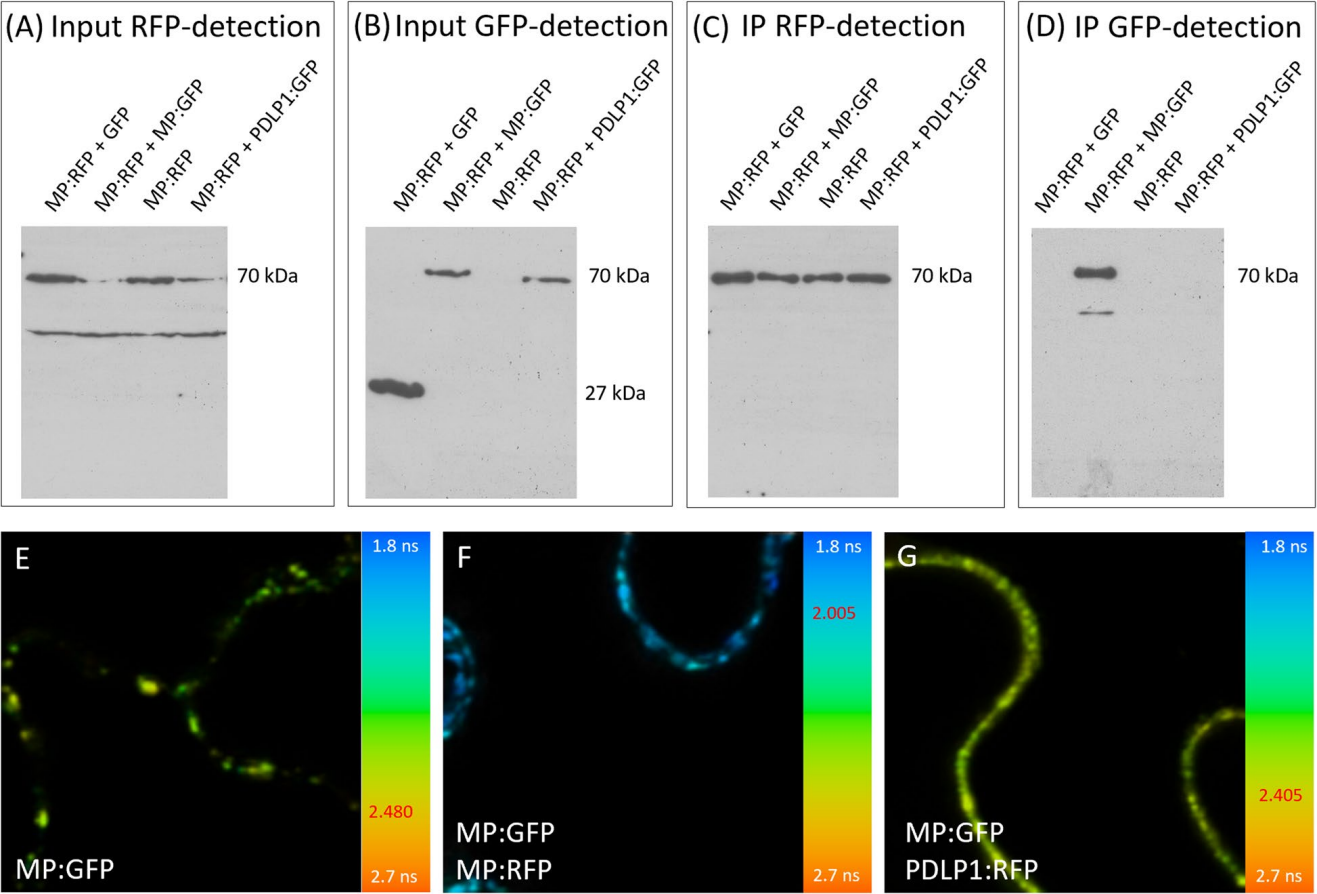
MP^JSBWMV^ self-interacts. **A-D** IP was conducted with leaf-material from *N. benthamiana* co-expressing MP^JSBWMV^:RFP and one of the GFP-tagged proteins, respectively, using RFP-trap antibodies. Western blots with anti RFP (**A**) and anti GFP (**B**) antibodies were conducted with crude protein extracts to demonstrate that all proteins were expressed. **A** MP^JSBWMV^:RFP was expected at approximately 70 kDa. The bands at approximately 50 kDa in the input sample likely represent unspecific antibody binding. **B** anti GFP antibodies detected the MP^JSBWMV^:GFP at approximately 70 kDa, PDLP1:GFP at approximately 65 KDa and GFP at approximately 27 KDa. **C** Western blots conducted with immunoprecipitated samples and probed with RFP-specific antibodies revealed the presence of MP^JSBWMV^:RFP (approximately 70 kDa) in all samples. **D** Western blots conducted with immunoprecipitated samples were probed with anti GFP antibodies, a band for MP^JSBWMV^:GFP (approximately 70 kDa) was detected while no bands for PDLP1:GFP (approximately 65 kDa), and for GFP (approximately 27 kDa) were visible. **E–G** Fluorescence lifetime of MP^JSBWMV^:GFP expressed in *N. benthamiana* epidermal cells was measured by FRET-FLIM. The images reveal fluorescence lifetime in a pseudo-color scheme, ranging from 1.8 ns (blue) to 2.7 ns (orange) as shown in the color-coded bar at the right hand side. The fluorescence lifetime of (**E**) MP^JSBWMV^:GFP expressed alone, (**F**) MP^JSBWMV^:GFP in the presence of MP^JSBWMV^:RFP, (**G**) MP^JSBWMV^:GFP in the presence of PDLP1:RFP

Several viral MPs were shown to self-interact [2, 41, 42]. This self-interaction may be important for viral ribonucleoprotein complex formation, or play a role in facilitating interactions with other viral factors. Therefore, we analyzed the ability of MP^JSBWMV^ to form multimers by co-immunoprecipitation using anti-RFP nano-traps and FRET-FLIM with a two-photon system, which measures time-correlated single-photon counting [31, 32]. We expressed MP^JSBWMV^:RFP alone or in combination with MP^JSBWMV^:GFP, GFP or PDLP1:GFP via agro-infiltration in *N. benthamiana* epidermal cells. The expression of all proteins was confirmed by western blots of crude plant extracts using specific antibodies against GFP and RFP (Fig. 3 A-B). MP^JSBWMV^:RFP successfully precipitated MP^JSBWMV^:GFP, but not GFP alone or PDLP1:GFP, a protein co-localizing with MP^JSBWMV^ at PD (Fig. 3 C-D). To further confirm the interaction, we conducted FRET-FLIM experiments in agroinoculated *N. benthamiana* epidermal cells co-expressing the proteins. Co-expression of MP^JSBWMV^:RFP strongly reduced the fluorescence lifetime of MP^JSBWMV^:GFP, indicating an interaction of the two proteins with a FRET-efficiency of 19.15% (Table 1, Fig. 3 E–F). Meanwhile, MP^JSBWMV^:GFP expressed with PDLP1:RFP showed only a modest reduction in fluorescence lifetime corresponding to a FRET-efficiency of 3.02% (Table 1, Fig. 3 E, G).

**Table 1:** Fluorescence lifetime values and percentage of FRET for FRET-FLIM experiments with MP^JSBWMV^:GFP in the presence of MP^JSBWMV^:RFP or PDLP1:RFP. The table shows fluorescence lifetime values for MP^JSBWMV^:GFP expressed alone or in the presence of MP^JSBWMV^:RFP or PDLP1:RFP. Lifetime values in ns, SD standard deviation in ns, N number of replicates, n the number of single measurements used to calculate the average. FRET-efficiency was calculated as FRET% and T-test (p-value 0.05) used to calculate statistical significance

Proteins	Localization	N	n	Lifetime [ns]	SD [ns]	% FRET	T-test
MP^JSBWMV^:GFP	PD	3	189	2.480	0.078	-	-
MP^JSBWMV^:GFP + MP^JSBWMV^:RFP	PD	3	339	2.005	0.126	19.15	2.2E-190
MP^JSBWMV^:GFP + PDLP1:RFP	PD	3	141	2.405	0.083	3.02	8E-16

### MP^JSBWMV^ binds to JSBWMV RNA1 and RNA2 with high affinity

**Fig. 4:**
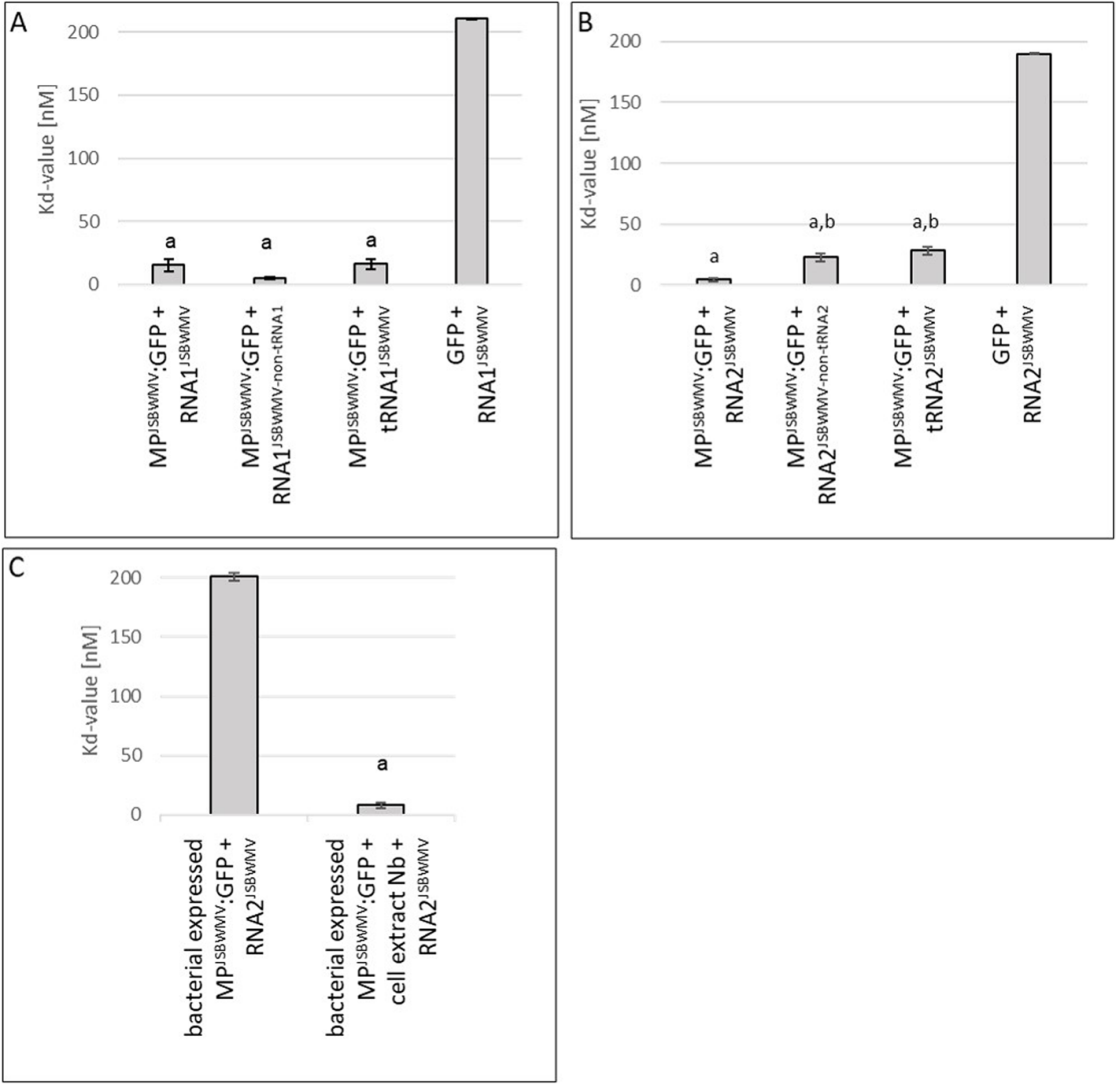
MP^JSBWMV^ can bind RNA1^JSBWMV^ and RNA2^JSBWMV^. *N. benthamiana* leaf crude extracts expressing MP^JSBWMV^ and GFP after agro-inoculation were used to analyze the binding of these proteins to (**A**) RNA1^JSBWMV^, (**B**) RNA2^JSBWMV^ and parts of the viral RNA with and without the tRNA-like structure, respectively. **a** significantly different to GFP control, b, significantly different to MP^JSBWMV^:GFP + RNA2^JSBWMV^. **C** binding affinity of MP^JSBWMV^:GFP purified from *E. coli* to RNA2^JSBWMV^ with and without plant extract from *N. benthamiana*. a, significantly different to MP.^JSBWMV^:GFP purified from bacteria without plant extract. **A**-**C** Statistical analysis was made with Tukey test (*p*-value < 0.05). Binding curves are presented in Fig. S2

The ability to bind RNA was described for several MPs [1, 2]. To test if MP^JSBWMV^ binds its own viral RNA, MP^JSBWMV^:GFP or GFP as control were expressed in *N. benthamiana* leaves. Leaf crude extract was used in microscale thermophoresis experiments with in vitro synthesized non-labelled JSBWMV RNA1 and RNA2. Typically, MST measurements are performed with purified proteins to retrieve direct interaction parameters. Obtaining pure and functional protein in needed quantities is often a major bottleneck. Nevertheless, interactions can be measured in complex sample environments like cell extracts as well, circumventing the need to purify the target protein. In contrast to purified proteins, obtained binding characteristics in cell extracts can significantly differ from purified protein sample showing stronger interactions than expected due to cooperative effects of present interaction partners within the cell lysate [43]. The dissociation constants (Kd) obtained for RNA binding of MP^JSBWMV^:GFP were 15.4 nM for RNA1^JSBWMV^ (Fig. 4A) and 4.5 nM for RNA2^JSBWMV^ (Fig. 4B) compared to Kd-values obtained for binding of GFP to JSBWMV RNAs (210.4 nM (RNA1^JSBWMV^, Fig. 4A) and 189.8 nM (RNA2^JSBWMV^, Fig. 4B)), thus indicating, that MP, but not free GFP, efficiently bound viral RNAs in crude leaf extract. Noteworthy, purified MP^JSBWMV^:GFP from *E. coli* showed a much weaker binding towards the RNA2^JSBWMV^, but affinity was restored when the purified MP^JSBWMV^:GFP was added into a cell extract from a non-expressing leaf (Fig. 4C). As recent studies indicated that tRNA-like structures may represent one determinant to mediate RNA mobility in plants [44, 45], we investigated whether the Kd values obtained for binding of viral RNAs differed in the presence or absence of the tRNA-like structure. The Kd-values for MP^JSBWMV^ binding to RNA1^JSBWMV^ and RNA2^JSBWMV^ without tRNA-like structure at the 3`-terminus were 5.5 nM (Fig. 4A) and 23.0 nM (Fig. 4B), respectively. MP^JSBWMV^ could bind the tRNA-like structure of RNA1^JSBWMV^ and RNA2^JSBWMV^ alone with Kd-values 16.2 nM (Fig. 4A) and 28.3 nM (Fig. 4B). These findings indicate that the tRNA-like structure is not essential for binding of MP^JSBWMV^ to the viral RNA^JSBWMV^.

### MP^JSBWMV^ binds to cellular RNAs with specific functions

We demonstrated that MP^JSBWMV^ can bind its own viral genome with high affinity. Previous studies have shown that RNA binding by MP is in general not sequence specific, as MPs from related viruses can also bind to the viral RNA genome of other viruses (e.g. [46, 47]). To explore which RNAs are bound by MP in a cellular environment, we conducted RIPs using *N. benthamiana* leaf extracts expressing MP^JSBWMV^:GFP or GFP (used as a control), with anti-GFP nano traps. Co-precipitated RNAs were analyzed by Illumina-sequencing. We identified 22 RNAs that were more than four-fold more abundant in the MP^JSBWMV^:GFP RIP compared to the GFP control RIP (Table 2). The list containing the most enriched RNAs revealed shared functions of the corresponding proteins, which were encoded by the RNAs (Table 2). Proteins encoded by these RNAs were involved in plant signaling (Table 2, blue) or involved in lipid metabolism and membrane modification (Table 2, yellow). Another group of proteins had chaperone functions and functions in protein degradation (Table 2, green). Interestingly, several of the proteins encoded by the immunoprecipitated RNAs have previously been implicated in virus infection.

**Table 2:**
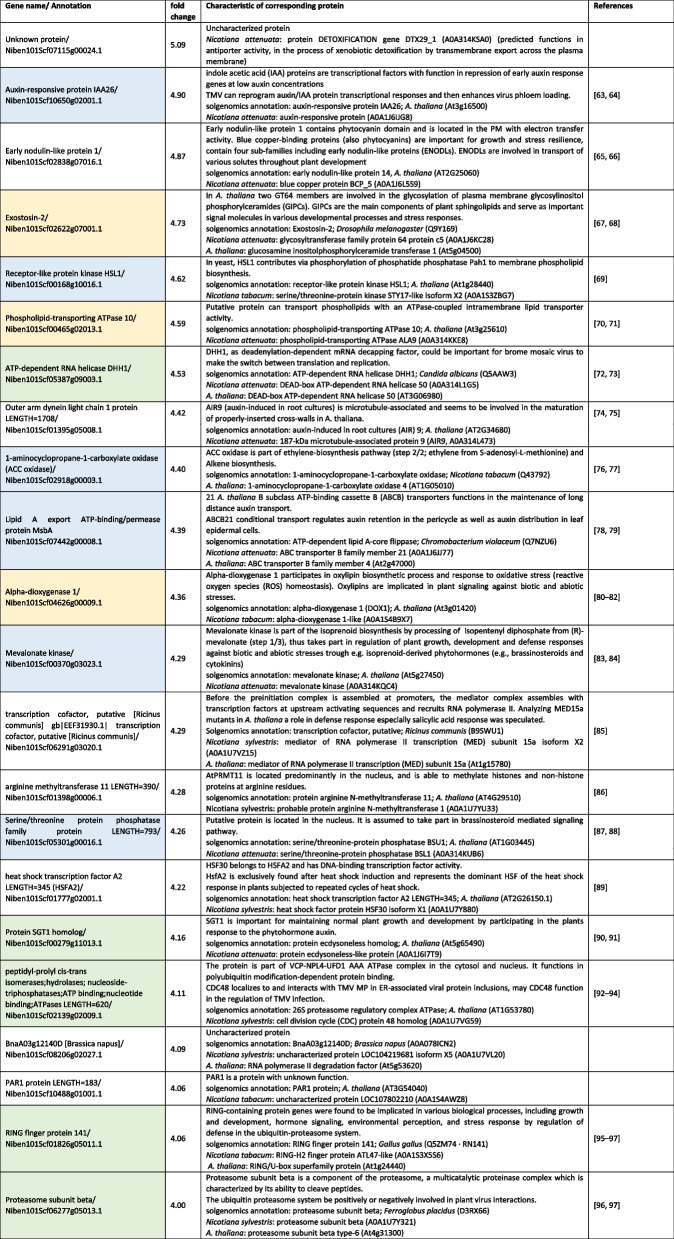
RNAs identified by RIP using MP^JSBWMV^ as target [63–97]

blue color, RNAs encoding proteins involved in signaling, yellow color, RNAs encoding proteins involved in lipid metabolism and membrane modification; green color, RNAs encoding proteins with chaperone functions and functions in protein degradation

## Discussion

To gain insight into the function of the JSBWMV movement protein, we here characterized the MP in terms of subcellular localization, role in facilitating virus transport through plasmodesmata, and the biochemical features self-interaction and binding to viral and host RNAs. The predicted MP encoded on RNA1 of JSBWMV shows similarities to the MPs of the 30 K family of other plant viruses [4]. Experiments with the MP of the closely related furovirus SBWMV revealed that MP fused N-terminally to GFP can move between wheat epidermal cells [14]. After biolistic bombardment clusters of cells expressing the GFP:MP^SBWMV^ were observed. Complementation of a movement-deficient TMV for short- as well as long-distance movement in *N. benthamiana* was also shown for MP^SBWMV^ [48]. Moreover, ectopically expressed MP^CWMV^ complemented a movement-deficient potato virus X [19]. Our results showing that MP^JSBWMV^ can complement movement of a movement-deficient TMV are consistent with the findings obtained for the other studied furoviruses and, importantly, demonstrate functionality of our fluorescent protein-tagged MP.

Whereas the three furoviral MPs studied to date are all promoting virus cell-to-cell movement, differences in the localization of the MP^JSBWMV^ compared to MP^SBWMV^ and MP^CWMV^ were observed. MP^SBWMV^ was shown to localize to the cytoplasm and the cell wall by immunogold labelling experiments [14]. GFP:MP^CWMV^ was reported to localize to PD and to ER-derived vesicles [19]. We found a localization of MP^JSBWMV^:GFP and MP^JSBWMV^:RFP to PD and PM microdomains. Localization to PD is a common feature for MPs, as they facilitate cell-to-cell movement through PD [1, 49]. We observed MP^JSBWMV^ localization in a patchwork pattern with remorin proteins. Co-localization experiments between proteins of the remorin family and viral MPs have been performed before, but also showed no perfect co-localization [50, 51]. Similarly to the cellular distribution of MP^JSBWMV^, the MP^FMV^ of fig mosaic virus (FMV) showed a localization to PD and PM microdomains [52, 53]. For MP^FMV^ the localization to PM microdomains and PD was functionally related and important for PD localization and cell-to-cell movement [52]. The localization of MP^CWMV^ to PD was shown to depend on the secretory pathway, as treatment with Brefeldin A and expression of a dominant negative SAR1 mutant retained the MP in the ER [19]. As we did not observe MP^JSBWMV^ in association with ER-derived structures, we did not test whether also MP^JSBWMV^ targeting to PD depended on the secretory pathway.

The ability of MP to from multimers is known to be important for their function; however, the exact role of MP multimers remains unclear [41]. For different viral MPs, self-interaction was demonstrated, including TMV, barley yellow dwarf virus-GAV or Abutilon mosaic virus [41, 54, 55]. We showed that also MP^JSBWMV^ forms multimers. To achieve cell-to-cell movement, MPs form protein complexes and bind to RNA [1, 49]. It is described that viral MPs bind preferentially single-stranded RNA in a sequence-independent manner [46, 47, 56, 57]. Specificity for the viral RNA genome is thought to be conferred by structural motifs in the MP and/or co-translational RNA binding by the MPs. Using MST, we found that MP^JSBWMV^ can bind its viral genome with high affinity. This affinity is dependent on potential further external factors that are present in the cell lysate and cannot be solely attributed to an intrinsic ability for the MP. Similar observations were made using cell lysates from mammalian cell cultures, thus showing that interactions with purified binding partners can significantly differ from interactions in cell lysates [43]. Currently, no RNA structure-specific recognition motif for MPs has been identified. Interestingly, for mobile endogenous mRNAs harboring a tRNA-like structure, abolishment of the mobility of mRNA transcripts was observed by deletion of the tRNA-like sequences and it was assumed that tRNA-like sequences are necessary and sufficient for long distance movement of certain mRNA molecules [44, 45]. Like many other viruses, furoviruses harbor tRNA-like sequences at the 3´ terminus of their genome [12, 13]. We therefore investigated whether the tRNA-like structure in the viral RNA influenced the binding affinity of the MP. Our results revealed that MP^JSBWMV^ binds the viral genome with comparable efficiency whether or not the tRNA-like structure is present. This suggests that the tRNA-like structure does not play a significant role in the recognition of the RNA genome by MP. Thus, either other structural motifs or signals may determine recognition by MP and the intercellular mobility of the viral RNA, or MP may not be the factor providing selectivity for those mobile viral RNAs. In this scenario, MP may be responsible for targeting RNA to PD and increasing PD SEL, while selectivity for the transported RNAs would be provided by a different factor.

To explore how viral MP co-opts host processes for efficient virus movement, we investigated the binding of host RNAs to MP^JSBWMV^. Interestingly, the functions of the proteins encoded by the subset of RNAs present in MP^JSBWMV^-complexes is consistent with the functions of proteins known to be co-opted during plus-strand virus replication [58, 59] and include proteins regulating signaling, lipid synthesis enzymes, chaperones and proteins involved in protein degradation. The subset of cellular RNAs identified as enriched in co-immunoprecipitation experiments using MP^JSBWMV^ vs. GFP is consistent with a model, which proposes a role of these MP-bound RNAs in supporting virus movement and replication. By binding specific RNAs, MP^JSBWMV^ may recruit them away from their cellular destination and/or contribute to endogenous RNA movement. Moreover, by binding specific RNAs and inducing their translation at the specific cellular site where they are required to fulfil a specific function may significantly contribute to the spatial and temporal coordination of cellular functions and virus infection cycle [60, 61]. In addition, by regulating key players in signaling, PD permeability may be modified to enhance virus movement [5]. Because viral MPs are often involved in the creation of membrane compartments for viral replication and cause membrane-rearrangements also in the absence of virus infection [2, 5], it appears possible that the RNA interaction partners of the viral MP identified here play a role during infection.

## Conclusions

We here provide data to demonstrate that MP^JSBWMV^ is a typical viral MP, which is functional in promoting virus cell-to-cell movement, capable of forming multimers, binds viral RNA without preference for tRNA-like structures, and shows cellular localization at PDs of plant cells. Identification of RNAs associated with the MP^JSBWMV^ indicates that it binds RNAs encoding proteins involved in signaling, membrane modification and protein folding and turnover. We conclude that by binding these RNAs, MPs may regulate their translation and consequently their spatio-temporal activity. Consistent with our findings, a meta-analysis of *Arabidopsis thaliana* regarding altered mRNAs upon viral infection found that the mRNAs of highly connected, central and modular genes are affected [62]. This suggests that viruses may preferentially interact with hub regulator genes during the infection process. It may also be possible that the virus binds these RNAs to regulate their cell-to-cell transport. Future studies will investigate the mobility of the identified RNAs and the function of the encoded proteins with respect to virus infection and movement. Recent advances in RNA imaging technology will help answering these questions. Moreover, future studies will investigate whether the subset of cellular RNAs bound by MP share specific features making these RNAs MP targets.

## Supplementary Information

Supplementary Material 1: Table S1: Primer sequences. Fig. S1: JSBWMV forms visible infection sites on *Chenopodium quinoa* leaves at 17 °C but not at 24 °C. Fig. S2: Microscale thermophoresis binding curves for MP^JSBWMV^:GFP or GFP, respectively to viral RNAs. Fig. S3: MP^JSBWMV^:RFP localizes to membrane rafts.

## Data Availability

Data is provided within the manuscript or supplementary information files. The raw sequencing data will be made available by the authors on request.

## Abbreviations

CP: Coat protein
CWMV: Chinese wheat mosaic virus
FLIM: Fluorescence lifetime imaging
FMV: Fig mosaic virus
FRET: Fluorescence resonance energy transfer
JSBWMV: Japanese soil-borne wheat mosaic virus
MP: Movement protein
MST: Microscale thermophoresis
PD: Plasmodesmata
PM: Plasma membrane
RIP: RNA-immunoprecipitation
SBWMV: Soil-borne wheat mosaic virus
SEL: Size exclusion limit
TMV: Tobacco mosaic virus
