# Supplementary material for: Studies on the Japanese soil-borne wheat mosaic virus movement protein highlight its ability to bind plant RNA
Source: Virol J. 2025 May 7;22:134. doi: 10.1186/s12985-025-02757-z (PMC12060307; doi:10.1186/s12985-025-02757-z)
Supplement: Supplementary file 1 — Supplementary Material 1: Table S1: Primer sequences. Fig. S1: JSBWMV forms visible infection sites on Chenopodium quinoa leaves at 17 °C but not at 24 °C. Fig. S2: Microscale thermophoresis binding curves for MPJSBWMV:GFP or GFP, respectively to viral RNAs. Fig. S3: MPJSBWMV:RFP localizes to membrane rafts. [file 12985_2025_2757_MOESM1_ESM.pdf]

Supplementary data

Studies on the Japanese soil-borne wheat mosaic virus movement protein highlight its ability to bind plant RNA

Authors: Claudia Janina Strauch[1], Nico Sprotte[1], Estefania Peña Lozano[2], Emmanuel Boutant[3,5], Khalid Amari[4], Steffen Ostendorp[2], Anna Ostendorp[2], Julia Kehr[2], Annette Niehl[1*]

[1]Julius Kühn Institute (JKI) - Federal Research Centre for Cultivated Plants, Institute for Epidemiology and Pathogen Diagnostics, Messeweg 11-12, 38104 Brunswick, Germany
[2]Institute of Plant Science and Microbiology, University of Hamburg, Ohnhorststr. 18, 22609 Hamburg, Germany
[3]Laboratory of Bioimaging and Pathologies, CNRS UMR 7021, Faculty of Pharmacy, University of Strasbourg, 74 route du Rhin - CS 60024, F-67401 Illkirch, Strasbourg, France
[4]Julius Kühn Institute (JKI) - Federal Research Centre for Cultivated Plants, Institute for Biosafety in Plant Biotechnology, Erwin-Baur-Str. 27, 06484 Quedlinburg, Germany
[5]Biotechnology and Cell Signaling, CNRS UMR7242, ESBS, University of Strasbourg, Bld Sébastien Brant, F-67412 Illkirch, Strasbourg, France

[*]**corresponding author:** annette.niehl@julius-kuehn.de

Table S1: Primer sequences

Primer name	Sequence 5´-3´	Determination
MP_GW_For1	GGG GAC AAG TTT GTA CAA AAA AGC AGG CTT AAA CAT GAC AAC TAA AGA TGT TTC	GATEWAY-cloning of MP^{JSBWMV}, addition of attB-sites
MP_GW_Rev1	GGG GAC CAC TTT GTA CAA GAA AGC TGG GTC CAC TAT GTT TTC AGA GTC	GATEWAY-cloning of MP^{JSBWMV}, addition of attB-sites
MP_fw_GG	TTA AGG TCT CGT TGC ATG ACA ACT AAA GAT GTT TCA AGT GAT TCC	GoldenGate cloning of MP^{JSBWMV}:GFP into *E.coli* expression plasmid pET28a
eGFP_rev_GG	TTA AGG TCT CGA AGC TTA CTT GTA CAG CTC GTC CAT GC	GoldenGate cloning of MP^{JSBWMV}:GFP into *E.coli* expression plasmid pET28a
eGFP_For	GGGG ACA AGT TTG TAC AAA AAA GCA GGC TTC ATG GTG AGC AAG GGC GAG	GATEWAY-cloning of free GFP
eGFP_Rev	GGGG AC CAC TTT GTA CAA GAA AGC TGG GTC TTA CTT GTA CAG CTC GTC CAT	GATEWAY-cloning of free GFP
MP_toRFP_NSp_For1	CCG AAG ATG CTC CCT GAA CA	Modification of pJS1
Bgl2_inRFP_NSp_Rev1	GAA GAT CTT TAG GCG CCG GTG G	Modification of pJS1
RNA2_for	GAT CTA ATA CGA CTC ACT ATA GTT GAG TGT TAA CTC TTC TTG	Full length RNA2 synthesis
RNA2_rev	CTC CGG TTC GGG GGG	RNA2 full length and tRNA-like structure-RNA2 synthesis
tRNA2_for	GAT CTA ATA CGA CTC ACT ATA TGT ATG TTC TGT TGA ACT ACT GT ATG	tRNA-like structure-RNA2 synthesis
tRNA1_for	GAT CTA ATA CGA CTC ACT ATA CAT AGT GTG ATT ATT ACT ATT ATG CTT T	tRNA-like structure-RNA1 synthesis
tRNA1_rev	CTC CGG TTC AGG GGG	tRNA-like structure-RNA1 synthesis
Non_tRNA1_for	GAT CTA ATA CGA CTC ACT ATA AGT CGT TAG ACT GCT TAA AG	non tRNA1-like-structure synthesis
Non_tRNA1_rev	TCA CAC TAT GTT TTC AGA GTC CAA	non tRNA1-like-structure synthesis
Non_tRNA2_for	GAT CTA ATA CGA CTC ACT ATA GTT GAG TGT TAA CTC TTC TTG	non tRNA2-like-structure synthesis
Non_tRNA2_rev	TCA CAG AGG TTT AGA CTT CCT T	non tRNA2-like-structure synthesis

Figure S1:

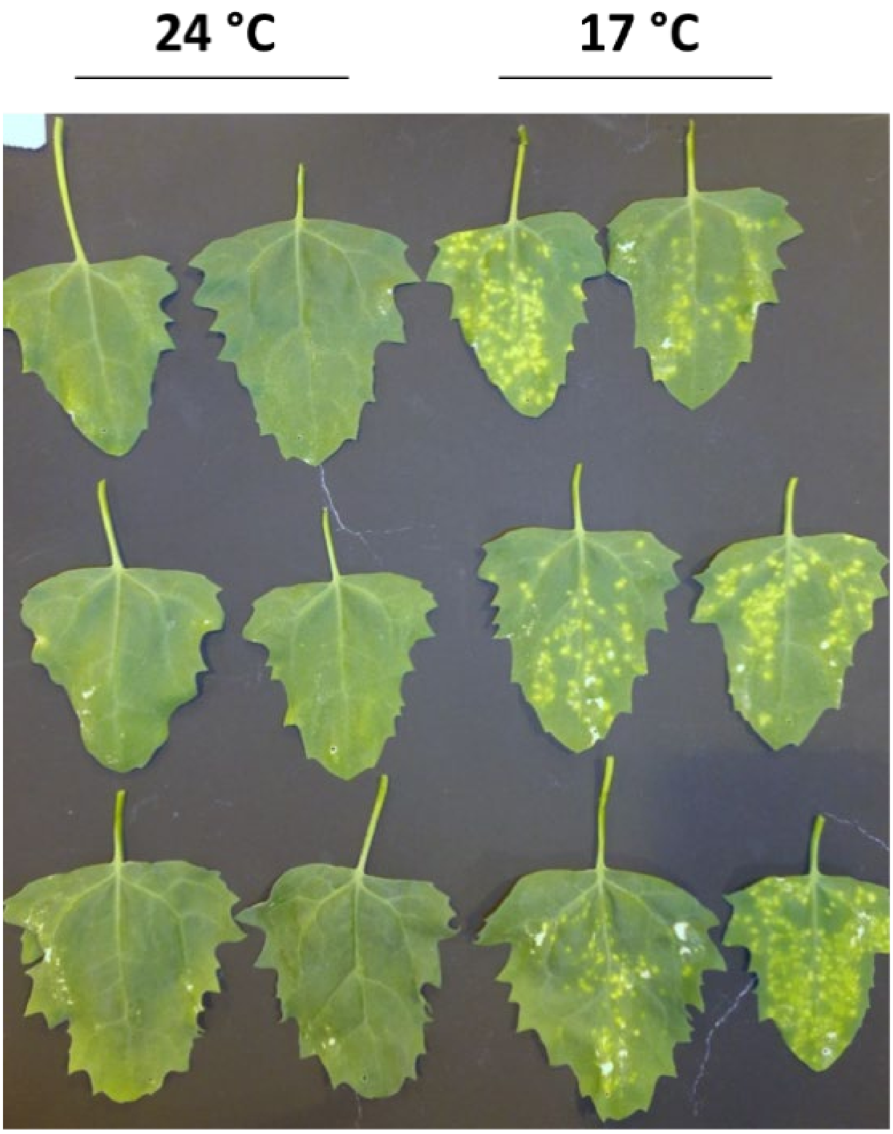

Figure S1. JSBWMV forms visible infection sites on *Chenopodium quinoa* leaves at 17 °C but not at 24 °C. *Chenopodium quinoa l*eaves of five to six-week-old plants were infected with RNA in-vitro transcribed from pJS1 and pJS2 cDNA clones (Yamamiya and Shirako, 2000) and kept in the greenhouse with 16h light and 8h dark cycles and at least 200 W/m^2 light intensity either at 24°C or at 17°C. Multicellular infection sites visible as chlorotic spots were only obtained at 17°C. Pictures were taken 12 dpi.

Figure S2:

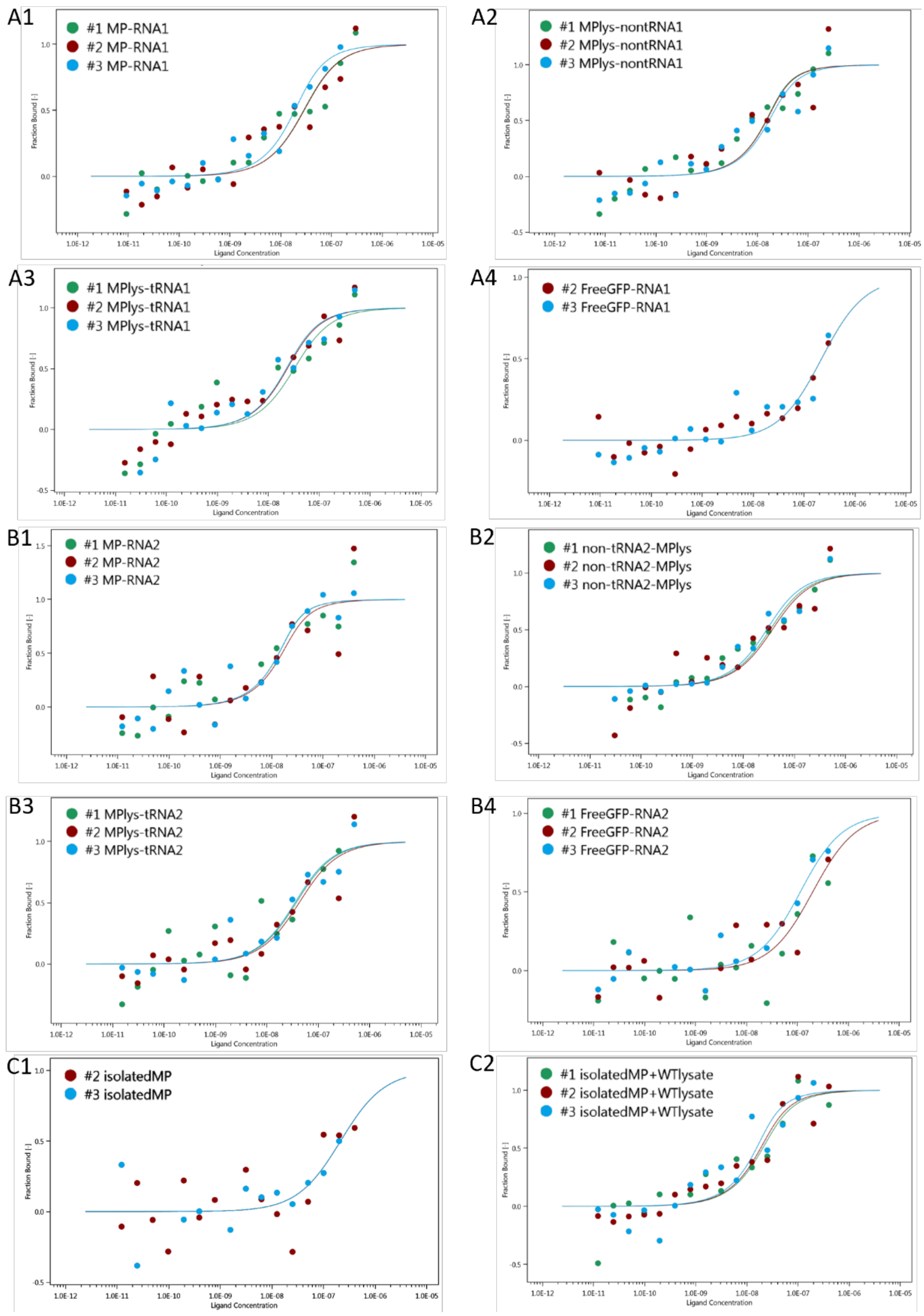

Figure S2: Microscale thermophoresis binding curves for MP^{JSBWMV}:GFP or GFP, respectively to viral RNAs

N. benthamiana leaf crude extracts expressing MP^{JSBWMV} and GFP after agro-inoculation were used to analyze the binding affinity of (**A1**) MP^{JSBWMV}:GFP to RNA1^{JSBWMV}, (**A2**) MP^{JSBWMV}:GFP to RNA1^{JSBWMV}-non-tRNA, (**A3**) MP^{JSBWMV}:GFP to tRNA1^{JSBWMV}, (**A4**) GFP to RNA1^{JSBWMV}, (**B1**) MP^{JSBWMV}:GFP to RNA2^{JSBWMV}, (**B2**) MP^{JSBWMV}:GFP to RNA2^{JSBWMV}-non-tRNA, (**B3**) MP^{JSBWMV}:GFP to tRNA2^{JSBWMV}, (**B4**) GFP to RNA2^{JSBWMV}, (**C1**) MP^{JSBWMV}:GFP isolated from bacteria to RNA2^{JSBWMV}, (**C2**) MP^{JSBWMV}:GFP to RNA2^{JSBWMV} isolated from bacteria complemented with *N. benthamiana* leaf extract.

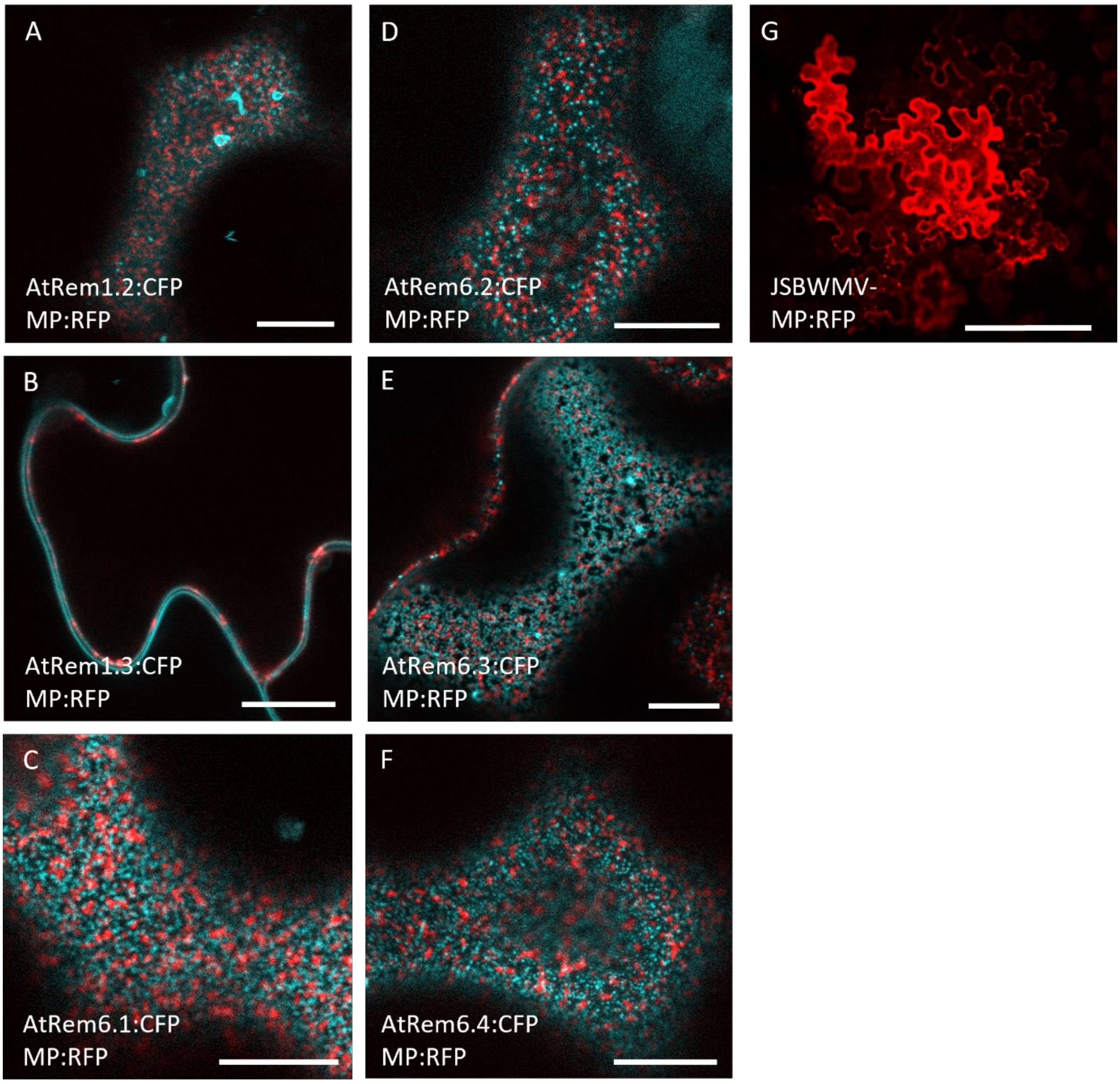

Figure S3: MP^{JSBWMV}:RFP localizes to membrane rafts.
(**A**-**F**) MP^{JSBWMV}:RFP was ectopically expressed in *N. benthamiana* epidermal cells via agroinoculation and co-expressed with different markers. (**A**-**F**) MP^{JSBWMV}:RFP (red) co-expressed with (**A**) AtREM1.2:CFP (cyan); (**B**) AtREM1.3:CFP (cyan); (**C**) AtREM6.1:CFP (cyan); (**D**) AtREM6.2:CFP (cyan); (**E**) AtREM6.3:CFP (cyan); (**F**) AtREM6.4:CFP (cyan), remorins and MP^{JSBWMV}:RFP localize in a patchy pattern at the cell periphery. The localizations do not overlap. Images taken two to five days post infiltration. Scale bars are 10 µm. (**G**) JSBWMV-MP:RFP infection site seven days after rub inoculation of RNA$^{JSBWMV\text{-}MP:RFP}$ into *N. benthamiana* epidermal cells. Scale bar is 200 µm.
